# Natural History Notes

**Published:** 1881-08

**Authors:** 


### NATURAL HISTORY NOTES.

*The Colors of Flowers.*—Hitherto it has been supposed that the colors of flowers were due to so many different materials, each color being a chemical combination having no relation with the others. But now, however, Professor Schuetzler, in a communication to the Vaudois Society of Natural Sciences, shows that, when the color of a flower is extracted by placing the latter in alcohol, the addition of an acid or alkali will give all the colors that plants exhibit. Flowers of pæony, for example, give when put into alcohol a violet-red liquid. If to this solution binoxalate of potassa ("salt of sorrell") be added the color becomes pure red. Soda causes it to change according to quantity used, to violet, blue, or green. In the latter case the green liquid appears red by transmitted light, just as a solution of chlorophyl (the green coloring matter of leaves) does. The sepals of pæony, which are green bordered with red, become entirely red when put into a solution of binoxalate of potassa. These changes of color, which may be obtained at will, may well be produced in plants by the same causes, since in all plants there are always acid or alkaline matters. Moreover, it is quite certain that the change from green to red observed in leaves in autumn is due to the action of the tannin which they contain on the chlorophyl. Consequently, without wishing to affirm it absolutely, Prof. Schuetzler believes that *a priori* there is in all plants but one coloring matter—chlorophyl—which, becoming modified by certain agents, gives all the tints that flowers and leaves exhibit. As for white flowers, it is well known that their want of color is due to the fact that their cells are filled with a colorless fluid, and that their opacity proceeds from the air contained in the interspaces.

When such flowers are placed under the receiver of an air-pump they are seen to lose their opacity and become transparent in measure as the air is exhausted.

